# Marrow Adipose Tissue: Skeletal Location, Sexual Dimorphism, and Response to Sex Steroid Deficiency

**DOI:** 10.3389/fendo.2017.00188

**Published:** 2017-08-04

**Authors:** Beata Lecka-Czernik, Lance A. Stechschulte, Piotr J. Czernik, Shermel B. Sherman, Shilong Huang, Amrei Krings

**Affiliations:** ^1^Department of Orthopaedic Surgery, University of Toledo Health Sciences Campus, Toledo, OH, United States; ^2^Department of Physiology and Pharmacology, University of Toledo Health Sciences Campus, Toledo, OH, United States; ^3^Center for Diabetes and Endocrine Research, University of Toledo Health Sciences Campus, Toledo, OH, United States

**Keywords:** bone marrow adipocytes, marrow adipose tissue, gonadal white adipose tissue, interscapular brown adipose tissue, beige fat, gene biomarkers, ovariectomy, orchiectomy

## Abstract

Marrow adipose tissue (MAT) is unique with respect to origin, metabolism, and function. MAT is characterized with high heterogeneity which correlates with skeletal location and bone metabolism. This fat depot is also highly sensitive to various hormonal, environmental, and pharmacologic cues to which it responds with changes in volume and/or metabolic phenotype. We have demonstrated previously that MAT has characteristics of both white (WAT) and brown (BAT)-like or beige adipose tissue, and that beige phenotype is attenuated with aging and in diabetes. Here, we extended our analysis by comparing MAT phenotype in different locations within a tibia bone of mature C57BL/6 mice and with respect to the presence of sex steroids in males and females. We report that MAT juxtaposed to trabecular bone of proximal tibia (pMAT) is characterized by elevated expression of beige fat markers including *Ucp1, HoxC9, Prdm16, Tbx1*, and *Dio2*, when compared with MAT located in distal tibia (dMAT). There is also a difference in tissue organization with adipocytes in proximal tibia being dispersed between trabeculae, while adipocytes in distal tibia being densely packed. Higher trabecular bone mass (BV/TV) in males correlates with lower pMAT volume and higher expression of beige markers in the same location, when compared with females. However, there is no sexual divergence in the volume and transcriptional profile of dMAT. A removal of ovaries in females resulted in decreased cortical bone mass and increased volume of both pMAT and dMAT, as well as volume of gonadal WAT (gWAT). Increase in pMAT volume was associated with marked increase in *Fabp4* and *Adiponectin* expression and relative decrease in beige fat gene markers. A removal of testes in males resulted in cortical and trabecular bone loss and the tendency to increased volume of both pMAT and dMAT, despite a loss of gWAT. Orchiectomy did not affect the expression of white and beige adipocyte gene markers. In conclusion, expression profile of beige adipocyte gene markers correlates with skeletal location of active bone remodeling and higher BV/TV, however bone loss resulted from sex steroid deficiency is not proportional to MAT expansion at the same skeletal location.

## Introduction

In recent years, a steady increase in research addressing the phenotype and function of marrow adipose tissue (MAT) has been observed. MAT is present in the bone marrow of all mammals and paradoxically its volume increases during both skeletal growth, which is associated with bone acquisition and sexual development, and aging, which is associated with bone loss and decline in gonadal activity. MAT can accumulate in the bone cavity in response to a variety of cues including hormonal (e.g., estrogen, parathyroid hormone), environmental (e.g., overnutrition, malnutrition, ambient temperature), and pharmacological (e.g., TZD and glucocorticoid therapies) [reviewed in Ref. (1)].

In healthy human adults, MAT constitutes up to 10% of total adipose tissues which translates to approximately 1 kg of mass. There is a significant difference in the volume, distribution, and appearance of marrow adipocytes between species. In humans, adipocytes represent up to 45% of cellular components in hematopoietic or red marrow, and up to 90% of adipocyte-rich yellow marrow. The yellow marrow gradually repopulates the marrow cavity of long bone during skeletal maturation and involution of hematopoietic marrow, and almost completely fills the cavity in the third decade of life (2). Marrow adipocytes in humans start to be visible in extremities (phalanges) of newborns and their postnatal accumulation is associated with a regression of hematopoiesis in these skeletal sites (3). The accumulation of MAT in humans progresses continuously during growth and aging in direction from appendicular to axial skeleton, and within the long bone from diaphysis to metaphysis (4). In contrast to humans, murine MAT accumulates in the direction from metaphysis toward diaphysis. MAT is present in distal tibia of C57BL/6 mice at very early age, but in proximal tibia it become visible with skeletal maturation which occurs around 3 months of age (5, 6).

It has been recently shown that MAT located in proximal tibia (pMAT) differs from MAT located in the distal part (dMAT) with regard to fatty acids composition and response to low temperature (7). The dMAT has higher fraction of unsaturated fatty acids when compared with pMAT, which may suggest different metabolic function of these two MAT depots. Moreover, pMAT, but not dMAT, responds to the cold exposure with decreasing in volume. On the other hand, caloric restriction increases MAT volume predominantly in proximal but not in distal location (7). The differences between these two MAT depots may be attributed to the heterogeneity of marrow adipocytes with respect to their origin and function (8).

One of the unique features of MAT is that it is simultaneously involved in the regulation of energy metabolism and bone homeostasis which may, at least in part, explain skeletal response to pathologic changes in energy balance (e.g., obesity, diabetes, caloric restriction, anorexia nervosa). MAT role in the regulation of energy balance comprises production of insulin sensitizing adiponectin at the levels that significantly contribute to the circulating pools of this adipokine especially in conditions of decreased peripheral fat mass due to either caloric restriction or anorexia nervosa (9, 10). On the other hand, MAT futile metabolic phenotype correlates positively with bone health and negatively with bone loss (5). BAT-like (energy production and dissipation) characteristics of MAT are compromised with diabetes and aging despite significant MAT expansion in the bone marrow (11). This supports a notion that there is a relationship between MAT metabolic profile and bone health. Indeed, marrow adipocytes have a capacity for conversion to beige-like phenotype either upon expression of specific transcriptional regulators, e.g., FoxC2 transcription factor (12), or as a result of manipulation with PPARγ transcriptional activity by either pharmacological use of selective agonists such as telmisartan (13), or by manipulation with PP5 phosphatase activity which controls PPARγ protein phosphorylation (14). Increase in the expression of beige fat markers in bone marrow MSCs was associated with increased expression of bone anabolic factors including Wnt10b, IGFBP2, and BMP4, and secretion to the growth media of pro-osteoblastic activities as assessed in co-cultture experiments (12, 14). Moreover, conversion of AD2 cells, representing marrow adipocyte cell line, to beige phenotype by either overexpression of FoxC2 (12) or by shRNA knockdown of PP5 (14) was associated with secretion of similar pro-osteoblastic activities strongly supporting the hypothesis that MAT with beige phenotype may possess beneficial for bone endocrine/paracrine activities.

Extramedullar adipose tissue serves several functions: it may store energy, it may dissipate energy, and it may regulate glucose metabolism in the endocrine manner. Fat tissue is categorized as of white (WAT), brown (BAT), or beige/brite phenotype which represent energy storing, dissipating, or both activities, respectively. The functional distinctions between different types of fat are reflected in a profile of expression of specific gene markers. With an exception of *Zic1* which is specifically expressed only in BAT, and *Tcf21* which is specifically expressed in WAT, the phenotype-specific expression of other markers is determined based on the relative levels, when compared with other fat types, and in combination with other markers. For example, *Ucp1* coding for uncoupling protein 1 is relatively high in BAT, low in WAT, but it can be induced in beige fat to the levels comparable in BAT and in beige fat it is associated with an increased expression of *Hoxc9, Prdm16, Tmem26, Dio2*, and/or *Tbx1* (15–17). As of now, MAT has not been characterized neither for its phenotype, nor function, nor profile of gene biomarkers expression.

Sex steroids are essential for adolescent bone growth and the maintenance of bone mass in adulthood. Postmenopausal bone loss or bone loss after removal of ovaries are the best examples of estrogen importance for bone metabolism in females. However, bone homeostasis in men also depends on estrogen, as mutations in the estrogen receptor or the aromatase affect maturation of skeleton and correlate with low bone mass (18). Similarly, removal of testes or low levels of testosterone in men with hypogonadism result in low bone mass, whereas high levels of circulating androgens in women with polycystic ovary syndrome correlate with high bone mass (19). Both estrogens and androgens regulate body composition and ultimately energy metabolism. Postmenopausal decrease in ovary function is associated with accumulation of WAT, especially in visceral location. On the cellular level, WAT expansion occurs due to an increased lipogenesis in adipocytes, the process which can be reverted by estrogen supplementation, indicating that lipogenesis and the associated gene expression are under the negative control of estrogen [reviewed in Ref. (20)]. On the molecular level, it has been shown that estrogen receptor and pro-adipocytic transcription factor and nuclear receptor PPARγ compete for the same steroid receptor coactivator Src-2, thus explaining at least in part their opposite activity in regards to adipocyte differentiation (21). In contrast, testosterone may either control lipolysis or lipogenesis in a depot-specific manner [reviewed in Ref. (20)]. In subcutaneous WAT of males and females, testosterone decreases lipolysis regulated by catecholamine, whereas *in vitro* testosterone decreases adipogenesis and adipocyte-specific gene expression. With respect to bone, there is a positive correlation between MAT accumulation in both appendicular and axial skeleton and postmenopausal bone loss and increased fractures (22–24). On the other hand, MAT volume decreases with estrogen replacement therapy giving a strong indication that MAT similarly as visceral WAT is under a negative control of estrogen (25). With respect to androgens however, there are no available systematic studies for the correlation between their levels and MAT volume.

In this manuscript, which is a part of a special issue on bone being an endocrine target and organ, we present studies on the correlation of MAT phenotype and bone mass, and MAT responsiveness to the sex steroids deficiency in males and females.

## Materials and Methods

### Animals

All analyzes were performed using C57BL/6 mice which were purchased from the Jackson Laboratories. Male and female mice were 4–6 months old, *n* = 4–8 per group. Animals were housed in a 12 h dark–light cycle and had free access to standard chow (Harlan Teklad 2016; Harlan, Haslett, MI, USA). Removal of ovaries and testes was accompanied by sham surgery in control animals. The effect of sex steroid deficiency on bone was analyzed 4 weeks after ovariectomy (OVX) or orchiectomy (ORX). To confirm successful removal of gonads, the weight of uterine or seminal vesicle was analyzed, respectively. The animal treatment and care protocols conformed to NIH Guidelines and the studies and the protocol were reviewed and approved by the University of Toledo Health Science Campus Institutional Animal Care and Utilization Committee.

### Microcomputed Tomography (mCT) Analysis of Bone Microarchitecture and Marrow Fat Content

Microcomputed tomography of the tibiae or femora was performed using the μCT-35 system (Scanco Medical AG, Bassersdorf, Switzerland), as previously described (26). Briefly, scans were performed at 70 kVp energy and 113 µA intensity settings and using 7 µm nominal voxel. Images of trabecular bone were segmented at 260 threshold values using per mille scale. The analysis of bone microstructure conformed to the recommended guidelines (27).

For lipid evaluation, decalcified bone specimens were stained for 1 h in solution containing 2% osmium tetroxide prepared in 0.1 M sodium cacodylate buffer pH 7.4, according to the protocol (26). Staining was carried-out in an exhaust hood and away from light due to osmium tetroxide toxicity and light sensitivity. Images of lipid depositions were acquired at 70 kVp and 113 µA settings and 12 µm nominal resolution. Image segmentation was done under global threshold condition by applying a gray scale threshold of 460 using per mille scale with filter set to sigma 1.2 and support 2.0. Lipid volumes were calculated directly from individual voxel volumes in 3-D reconstructions.

### Gene Expression Analysis Using Quantitative Real-time PCR

Depending on the experiment, total RNA was isolated either from entire tibia bone or proximal half and distal half, using TRIzol (Sigma-Aldrich, St. Louis, MO, USA). Before RNA isolation, bone periosteal surface was thoroughly cleaned from any remaining muscle, adipocyte, and connective tissue. One microgram of RNA was converted to cDNA using Verso cDNA synthesis kit (Thermo Fisher Scientific, Waltham, MA, USA). PCR amplifications were performed using TrueAmp SYBR Green qPCR SuperMix (Smart Bioscience, Maumee, OH, USA) and processed using StepOne Plus System (Applied Biosystems, Carlsbad, CA, USA). The thermocycling protocol consisted of 10 min step at 95°C, 40 cycles of 15 s at 95°C, and 60 s at 60°C, one final 10 min extension step at 72°C, followed by a melting curve step ranging from 60 to 95°C to confirm the specificity of amplification products. Primers were designed using OligoPerfect Designer (Thermo Fisher Scientific, Waltham, MA, USA). Primer sequences are listed in Table 1. Gene expression levels were assessed by using the comparative ΔΔCT method and *18S* RNA levels for normalization of all samples. An approximation of adipocyte-specific gene expression was calculated as a ratio of relative quantity (RQ) of given gene to the RQ of *Fabp4* or *Adiponectin* in the same sample. A relative expression between samples of a given gene was calculated by comparing its expression to one arbitrary chosen sample.

**Table 1:** Primer sequences used for real-time PCR.

Gene	F-primer	R-primer
*18S*	TTCGAACGTCTGCCCTATCAA	ATGGTAGGCACGGCGACTA
*Adip*	GGCCGTTCTCTTCACCTACG	TGGAGGAGCACAGAGCCAG
*Fabp4*	GCGTGGAATTCGATGAAATCA	CCCGCCATCTAGGGTTATGA
*Tbx1*	GGCAGGCAGACGAATGTTC	TTGTCATCTACGGGCACAAAG
*HoxC9*	GCAGCAAGCACAAAGAGGAGAAG	GCGTCTGGTACTTGGTGTAGGG
*Ucp1*	GGATGGTGAACCCGACAACT	AACTCCGGCTGAGAAGATCTTG
*Prdm16*	CCTAACTTTCCCCACTCCCTCTA	GCTCAGCCTTGACCAGCAA
*Dio2*	AAATGACCCCTTTGGTTTCC	TTC CCC ATT ATC CCT TTT CC
*Cidec*	AGGGAGGGACCTTAGGGAAT	CCAAGTCCAGCTTGGTGAAT
*Zic1*	AAC CTC AAG ATC CAC AAA AGG A	CCT CGA ACT CGC ACT TGA A
*Tmem26*	ACC CTG TCA TCC CAC AGA G	TGT TTG GTG GAG TCC TAA GGT C
*Tcf21*	CAT TCA CCC AGT CAA CCT GA	TTC CTT CAG GTC ATT CTC TGG
*Lep*	ATT TCA CAC ACG CAG TCG GTA T	GGT GAA GCC CAG GAA TGA AG
*RankL*	CCTGAGGCCCAGCCATTT	CTTGGCCCAGCCTCGAT

### Statistical Analysis

Statistical analysis of data consisted of unpaired two-tailed Student’s *t*-test after passing Shapiro–Wilk normalization test. All data shown represent mean and SD. A *p* < 0.05 was considered statistically significant.

## Results

### Morphological Appearance and Phenotypic Polarity of MAT in Proximal and Distal Tibia

**Figure 1:**
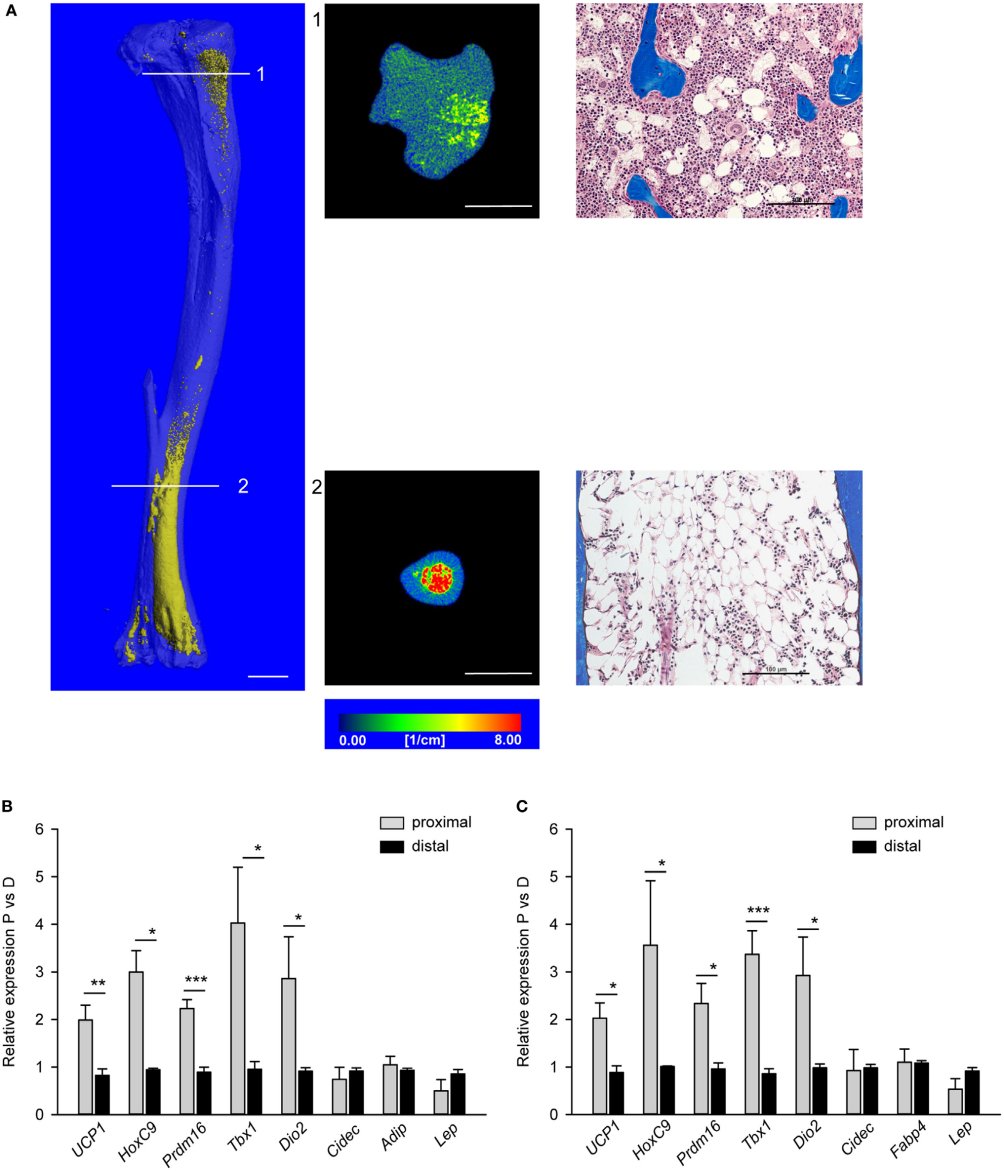
Morphology and polarity of tibia marrow adipose tissue (MAT). **(A)** (Left) Longitudinal microcomputed tomography (mCT) rendering of decalcified tibia bone stained with osmium tetroxide to visualize lipids (yellow). (Middle) Cross-sectional mCT renderings to visualize MAT distribution and density in proximal and distal tibia. Color scale indicates material density. The unit is a linear attenuation coefficient μ. Scale bars represent 1 mm. (Right) Representative longitudinal sections of proximal and distal non-decalcified tibia specimens stained with Masson’s Trichrome Stain (20× magnification). Scale bars represent 100 µm. **(B)** Gene expression profile of fat metabolic markers in proximal and distal tibia normalized to *Fabp4* expression. **(C)** The same gene expression analysis as in **(B)** but were normalized to the levels of *Adiponectin* expression. Analysis was performed on four tibia bone isolated from four males (6 months old). *p < 0.05; **p < 0.01; ***p < 0.001.

Morphology of MAT located in proximal and distal tibia was analyzed in decalcified bone specimens stained with osmium tetroxide to visualize lipid-filled cell distribution using mCT and in non-decalcified histological specimens. As showed in Figure 1A, MAT juxtaposed to trabecular bone in proximal tibia consists of adipocytes dispersed between trabeculae (cross-section 1 and adjacent histological image), whereas MAT located in distal tibia consists of densely packed adipocytes adjacent to the bone endosteal surface (cross-sections 2 and adjacent histological image).

We previously showed that WAT and BAT gene markers expression was detectable in tibia bone homogenates and their levels change in conditions of aging and diabetes which are associated with alterations in energy metabolism and bone remodeling (11). These, together with characteristic MAT distribution in murine tibia bone, led us to hypothesize that pMAT, which is in close proximity to the remodeling bone, have different gene markers expression profile from dMAT which is located close to the not remodeling bone. Therefore, we analyzed separately proximal and distal tibia for the profile of expression of gene markers specific for beige, brown, or white adipocyte (15–17). Table 2 provides a list of markers used in our analysis and their description.

**Table 2:** Gene biomarkers for brown, beige, and white adipocytes, and their relative expression.

Gene marker	Gene name/function in adipocytes	Relative expression			Primers used for real-time PCR
		BAT	Beige	WAT	
Zic1	Zic family member 1/transcription factor, C2H2-type zinc finger protein	+++	0	0	F: AAC CTC AAG ATC CAC AAA AGG A, R: CCT CGA ACT CGC ACT TGA A
Ucp1	Uncoupling protein 1/mitochondrial proton leak and heat production	+++	+ (+++ upon stimulation)	0	F: GGA TGG TGA ACC CGA CAA CT, R: AAC TCC GGC TGA GAA GAT CTT G
Hoxc9	Homeobox C9/transcription factor	0	+++	++	F: GCA GCA AGC ACA AAG AGG AGAAG, R: GCG TCT GGT ACT TGG TGT AGG G
Prdm16	PR domain containing 16/transcriptional corregulator of brown/beige adipocyte differentiation	+++	++	+	F: CCT AAC TTT CCC CAC TCC CTC TA, R: GCT CAG CCT TGA CCA GCA A
Tmem26	Transmembrane protein 26/multiple transmembrane helixes, surface marker for beige adipocytes	+	+++	++	F: ACC CTG TCA TCC CAC AGA G, R: TGT TTG GTG GAG TCC TAA GGT C
Tbx1	T-Box 1/transcription factor	0	+++	+	F: CCA TGA TAT CGG AAC AGA GAT G, R: ATG GCA GGA AAC ATC CTC CT
Dio2	Type II iodothyronine deiodinase/activates thyroid hormone by converting T4 to T3, regulator of thermogenesis	+++	++	+	F: AAA TGA CCC CTT TGG TTT CC, R: TTC CCC ATT ATC CCT TTT CC
Cidec (Fsp 27)	Cell death-inducing DFFA-like effector C/lipid droplet formation, restriction of lipolysis, apoptosis	ND	ND	+++	F: AGG GAG GGA CCT TAG GGA AT, R: CCA AGT CCA GCT TGG TGA AT
Tcf21	Transcription factor 21/basic helix-loop-helix family of transcription factors	0	0	+++	F: CAT TCA CCC AGT CAA CCT GA, R: TTC CTT CAG GTC ATT CTC TGG
Adipoq	Adiponectin/insulin sensitizing adipokine, which levels negatively correlate with fat mass	+	+++	+++	F: GGC CGT TCT CTT CAC CTA CG, R: TGG AGG AGC ACA GAG CCA G
Lep	Leptin/“Satiety” adipokine, which levels positively correlate with fat mass	+	+	+++	F: ATT TCA CAC ACG CAG TCG GTAT, R: GGT GAA GCC CAG GAA TGA AG
Fabp4 (aP2)	Fatty acids binding protein 4/fatty acids uptake, transport, and metabolism	+++	+++	+++	F: GCG TGG AAT TCG ATG AAA TCA, R: CCC GCC ATC TAG GGT TAT GA

*References (15–17). Key to relative units of expression levels as compared between all three fat depots*.

*+++, high; ++, moderate; +, low; 0, not expressed; ND, no data; BAT, interscapular brown adipose tissue; beige, inguinal adipose tissue representing beige fat; WAT, gonadal white adipose tissue*.

The homogenates of either proximal or distal tibia halves were used as RNA source for synthesis of cDNA templates for real-time quantitative PCR. Since we did not analyze purified homogeneous MAT cell populations, the expression of tested gene markers was normalized to the expression of adipocyte-specific gene markers, either *Fabp4* (Figure 1B) or *Adiponectin* (*Adip*) (Figure 1C). As shown, the expression of beige adipocyte gene markers, including *Ucp1, HoxC9, Prdm16, Tbx1*, and *Dio2*, was higher in proximal than distal tibia regardless whether *Fabp4* or *Adip* was used for normalization. This validated the use of surrogate markers for normalization of phenotype-specific gene expression in heterogeneous tissue such as bone marrow. Interestingly, we did not detect the BAT-exclusive marker *Zic1*, nor beige-specific marker *Tmem26*, nor WAT-exclusive marker *Tcf21*, neither in pMAT nor in dMAT. These imply that marrow adipocytes may differ from peripheral adipocytes at least in regards to expression of these markers.

The results presented in Figure 1 suggest that MAT with beige-like phenotype of energy dissipation is located in marrow cavity where bone remodeling is active, whereas MAT with white-like phenotype of lipid storing is located in non-remodeling bone of distal tibia. The correlation of MAT metabolic profile with location in marrow cavity suggests that MAT may contribute, either in positive or negative manner, to the bone marrow environment supporting bone remodeling.

### Beige Gene Markers Expression Is Higher in Proximal Tibia of Males and Correlates with Higher Trabecular Bone Mass (BV/TV)

**Figure 2:**
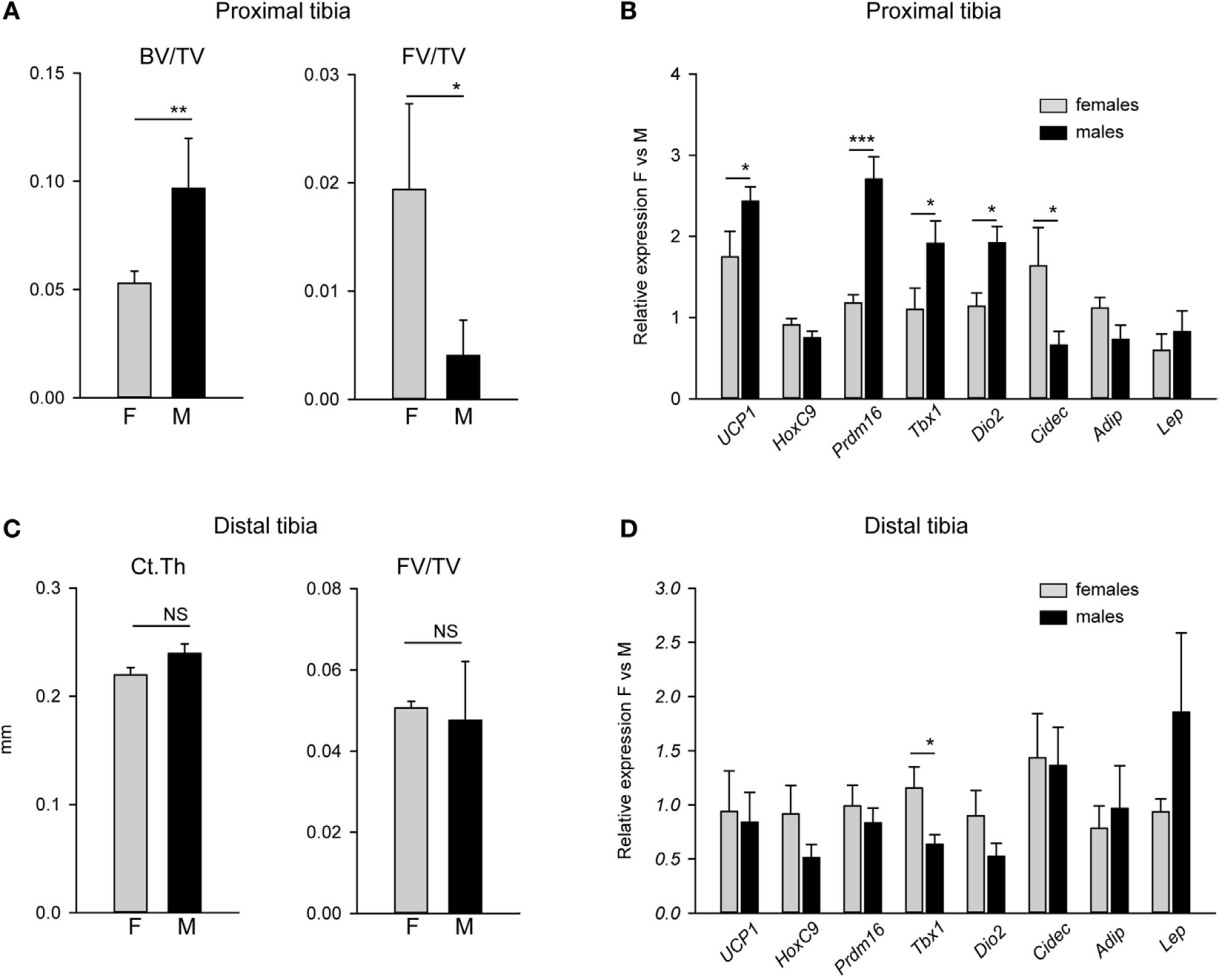
Comparison of bone mass and expression of adipocyte metabolic gene markers in tibia bone of males and females. **(A)** Trabecular bone mass and fat volume (FV/TV) in proximal tibia of 4 months old males (M) and females (F). **(B)** Analysis of expression of adipocyte-specific gene markers in proximal tibia of the same mice as in **(A)**. Relative expression of gene markers was normalized to *Fabp4* expression in the same samples. **(C)** Cortical thickness measured in tibia midshaft and FV/TV in distal tibia. **(D)** Analysis of expression of adipocyte-specific gene markers in distal tibia. Relative expression of gene markers was normalized to *Fabp4* expression in the same samples. All analysis were performed on four tibia bones isolated from four different animals per group. *p < 0.05; **p < 0.01; ***p < 0.001.

Since male mice, similarly to humans, have higher bone mass than females, we tested whether there is a relationship between MAT metabolic gene expression profile, bone mass, and sex. When compared with females, higher trabecular bone volume in proximal tibia of males was inversely related to fat volume (FV/TV) in the same location (Figure 2A). The BV/TV in males was twice as that of females, while pMAT volume in males was approximately 10-fold lower than in females. As shown in Figure 2B, the expression of beige fat markers in pMAT was higher in males than in females. These differences included 2.5-fold higher expression of *Prdm16*, almost twofold higher expression of *Tbx1* and *Dio2*, and elevated expression of *Ucp1*, but no difference in the expression of *HoxC9*. With respect to WAT-type markers, the expression of *Cidec* which promotes lipid droplets formation was decreased in males, whereas expression of *Adip* and leptin (*Lep*) were at the same levels in pMAT derived from males and females. In contrast, dMAT volume and markers expression did not differ between males and females and correlated with a lack of differences in bone cortical thickness (CtTh) (Figures 2C,D).

### The Effect of Sex Steroids Deficiency on MAT Volume and Phenotype-Specific Gene Markers Expression

To test the possibility that MAT volume and phenotype are regulated by sex steroids, we used mice models of either estrogen deficiency by surgical removal of ovaries or testosterone deficiency by surgical removal of testes. Four weeks after surgery, animals were analyzed for body parameters, bone mass, pMAT and dMAT volume, and expression of metabolic gene markers.

**Figure 3:**
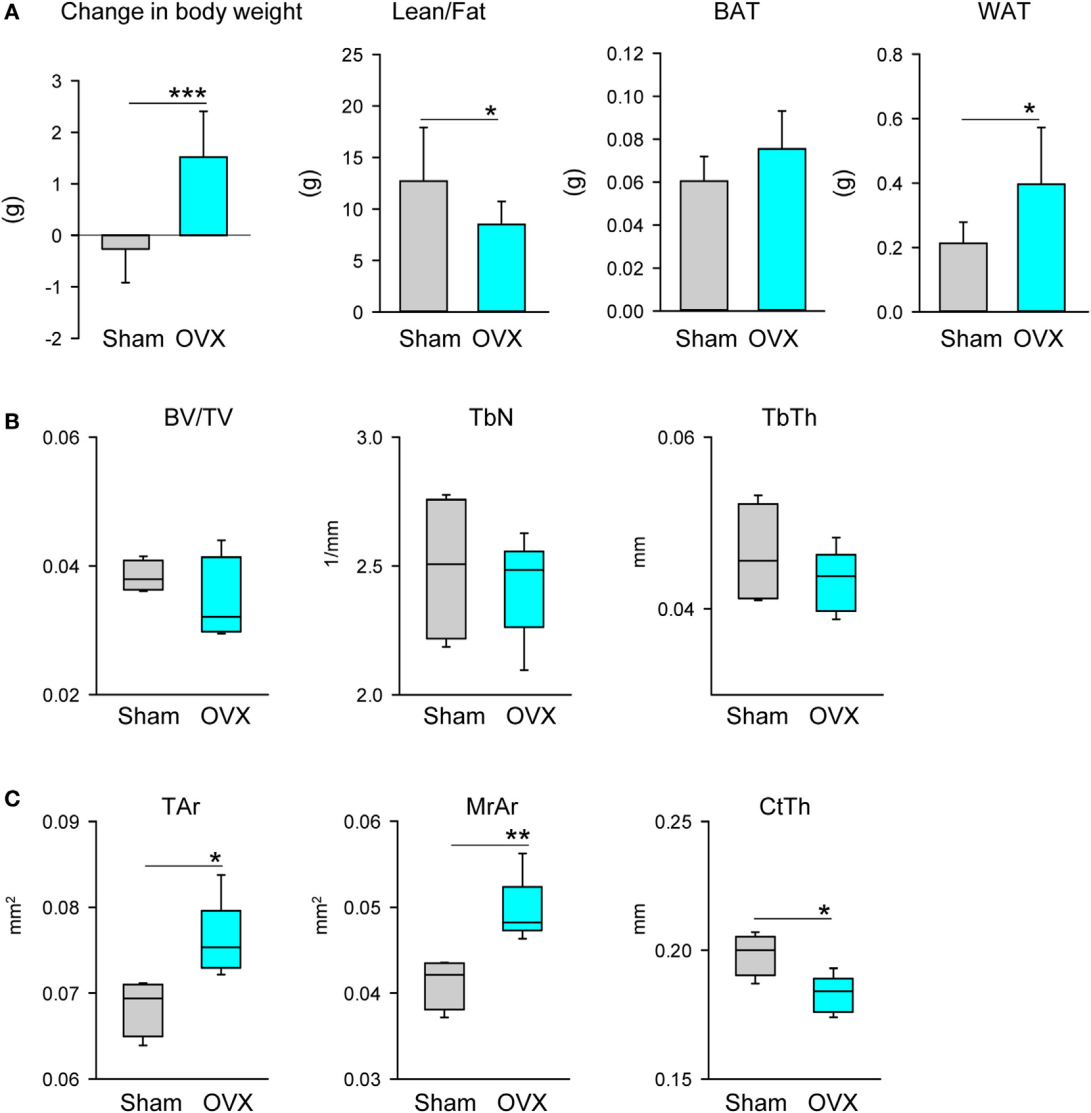

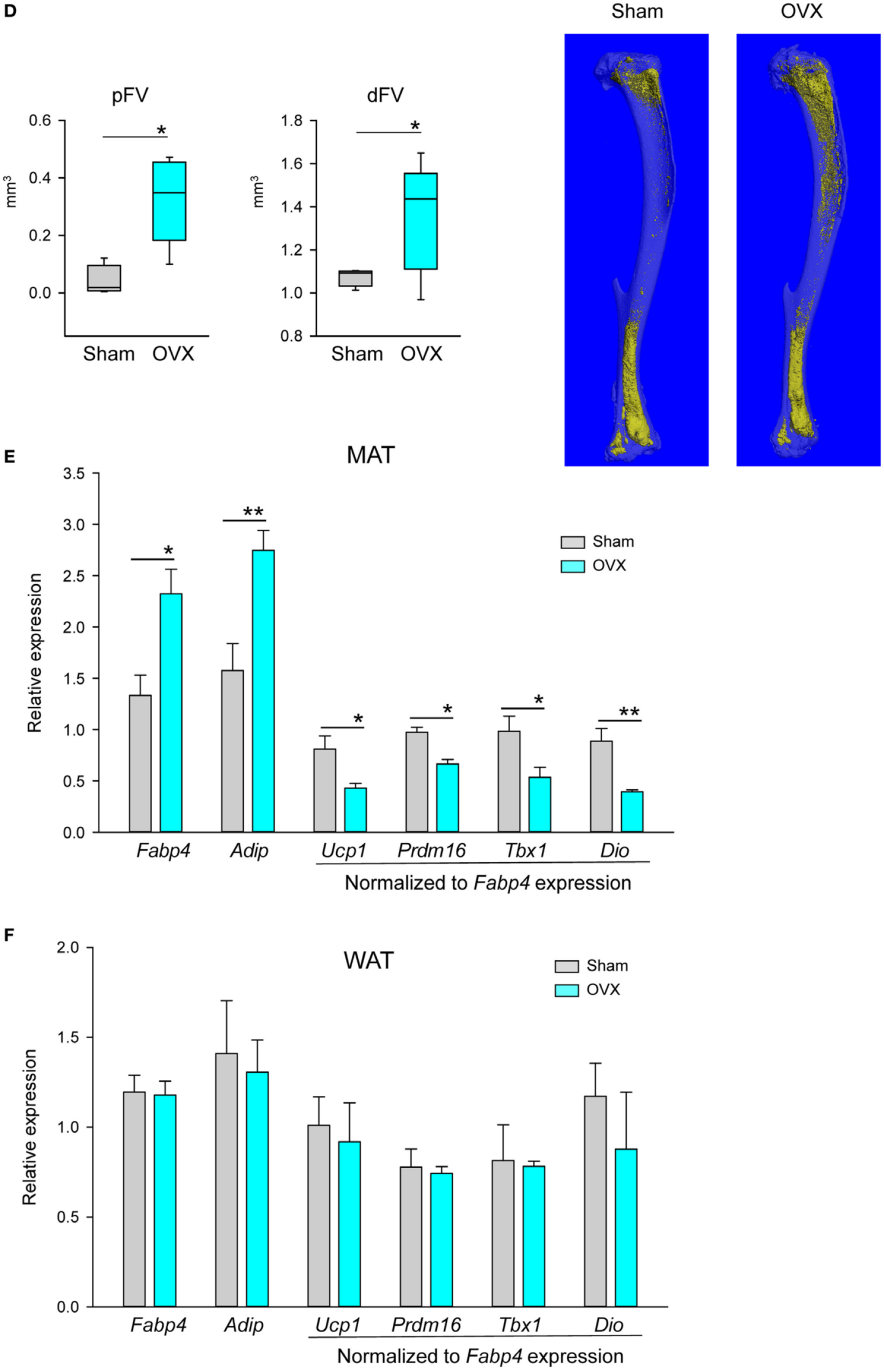
Effect of ovariectomy (OVX) on body composition, bone mass, and profile of gene markers expression. Mice (4 months old, *n* = 8 per group) underwent either Sham surgery or OVX and were sacrificed 4 weeks after surgery. **(A)** Body weight change, body composition measured with NMR, and weights of gonadal WAT (gWAT) and interscapular BAT were measured at the end of experiment. **(B)** Analysis of trabecular bone in proximal tibia. BV/TV, trabecular bone mass; TbN, trabeculae number; TbTh, trabeculae thickness. **(C)** Cortical bone mass measured in midshaft tibia. Tar, total area including bone area and marrow cavity area; MrAr, marrow cavity area; CtTh, cortical thickness. **(D)** Marrow fat volume in proximal and distal tibia, and microcomputed tomography images of marrow adipose tissue in tibia bone stained with osmium tetroxide. **(E)** Profile of gene expression markers in proximal tibia. **(F)** Profile of gene expression markers in gWAT isolated from the same animals as in **(D)**. In both panels, expression of *Ucp1, Prdm16, Tbx1*, and *Dio2* was normalized to *Fabp4* expression in the same sample (*n* = 4 tibia bones or four gWAT from four different animals per group). *p < 0.05; **p < 0.01; ***p < 0.001.

As expected, lack of estrogen led to an increase in body weight associated with an increase in fat and a decrease in lean mass, as measured with NMR (Figure 3A). An expansion of fat mass was seen in the gonadal WAT (gWAT) but not in the interscapular BAT (iBAT) (Figure 3A). Bone loss in tibia after OVX occurred mainly in the cortical and not in the trabecular compartment (Figures 3B,C), which is comparable with other studies showing that in adult (4–6 months old) C57BL6 mice OVX did not affect BV/TV in proximal tibia (28, 29). In diaphysis, loss of cortical bone was associated with an increase in bone perimeter, an increase in area of marrow cavity, and a decrease in CtTh, consistent with increased endosteal bone resorption (Figure 3C). Loss of ovary hormones was accompanied with prominent accumulation of adipocytes in the bone marrow of proximal and distal tibia (Figure 3D). An analysis of markers of beige phenotype, which expression correlated with differences in the bone mass between males and females as shown in Figure 2B, is presented in Figure 3E. Consistent with increased fat volume in proximal tibia the expression of *Fabp4* and *Adip* was significantly increased, whereas expression of *Ucp1, Prdm16, Tbx1*, and *Dio2* was decreased after adjustment to *Fabp4* expression. The expression of these markers was not affected in distal tibia of ovariectomized animals (not shown). Since gWAT similarly to MAT responded to estrogen deficiency with an increase in mass, we have tested this depot for any changes in gene expression which would indicate change in adipocyte metabolism. As shown in Figure 3F, similarly as in dMAT there was no alteration in the expression of tested markers including *Fabp4* and *Adip*, markers of *de novo* adipocyte differentiation.

**Figure 4:**
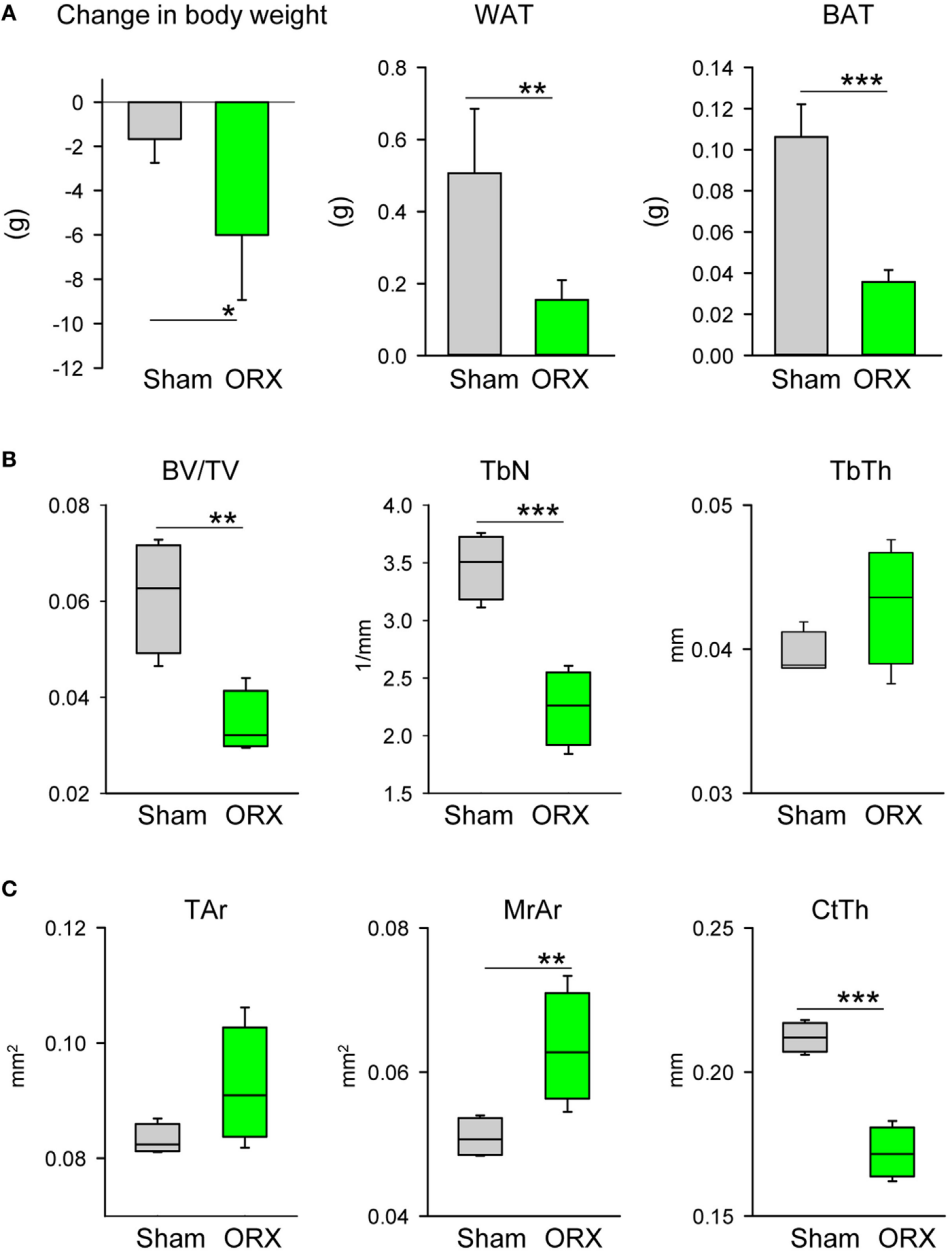

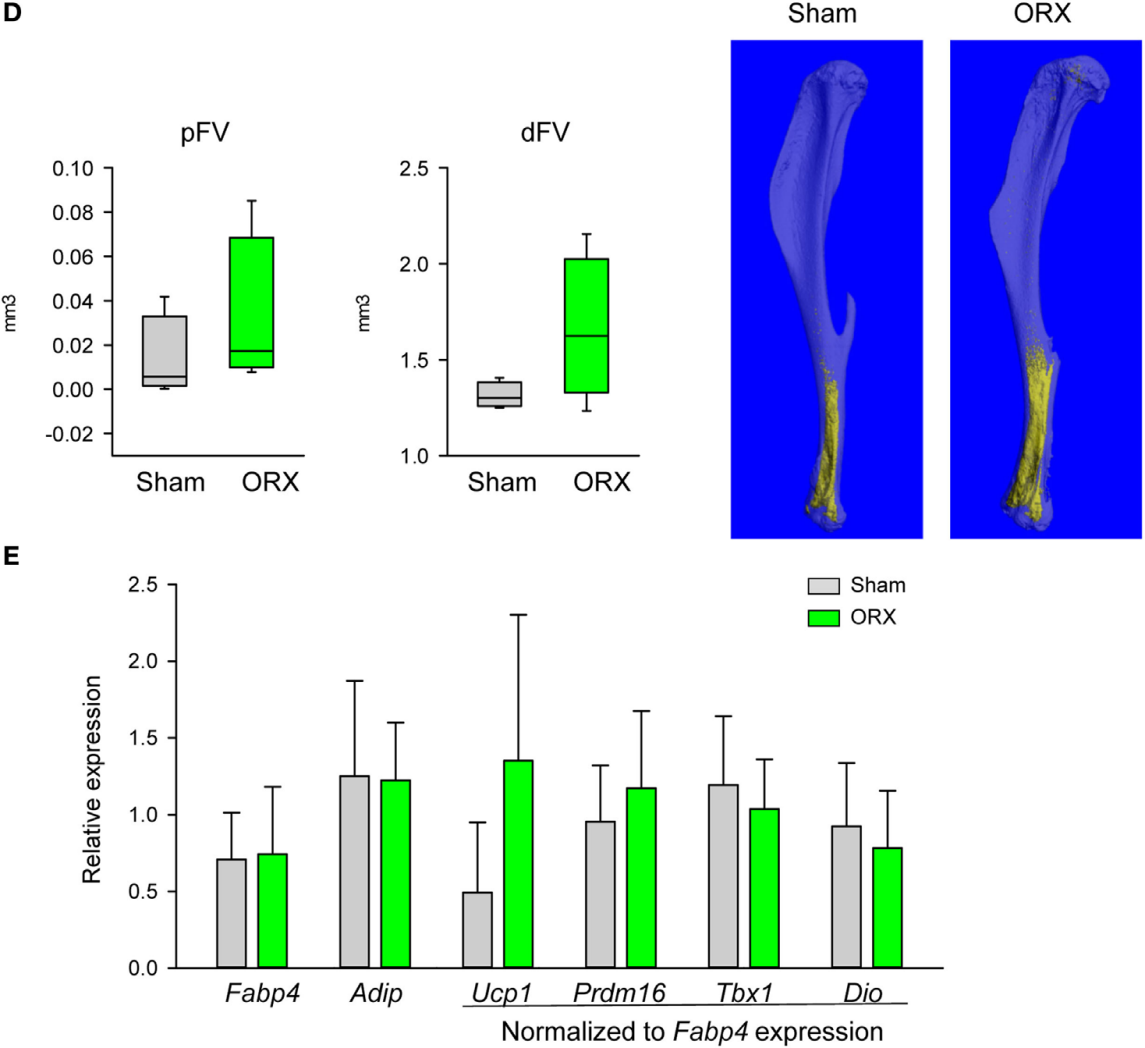
Effect of orchiectomy (ORX) on body composition, bone mass, and profile of gene markers expression. Mice (4 months old, *n* = 4 per group) underwent either Sham surgery or ORX and were sacrificed 4 weeks after surgery. **(A)** Body weight change, and weights of gonadal WAT and interscapular BAT measured at the end of experiment. **(B)** Analysis of trabecular bone in proximal tibia. BV/TV, bone mass; TbN, trabeculae number; TbTh, trabeculae thickness. **(C)** Cortical bone mass measured in midshaft tibia. Tar, total area measured including bone and marrow cavity; MrAr, area of marrow cavity; CtTh, cortical thickness. **(D)** Marrow fat volume in proximal and distal tibia, and microcomputed tomography images of marrow adipose tissue in tibia bone stained with osmium tetroxide. **(E)** Profile of gene expression markers in proximal tibia. The expression of *Ucp1, Prdm16, Tbx1*, and *Dio2* was normalized to *Fabp4* expression in the same sample (*n* = 4 tibia bones from four different animals per group). *p < 0.05; **p < 0.01; ***p < 0.001.

In contrast to the estrogen deficiency in females, lack of testosterone in males led to wasting phenotype signified by a decrease in body weight and decrease in mass of both gWAT and iBAT (Figure 4A). ORX led to an extensive loss of trabecular and cortical bone (Figures 4B,C). Trabecular bone loss was characterized with a decrease in the number of trabeculae (Figure 4B), whereas cortical bone loss was associated with expansion of marrow cavity and decrease in CtTh indicating increased endosteal bone resorption (Figure 4C). Despite a decrease in gWAT and iBAT mass, the tendency to MAT expansion in proximal and distal tibia was observed, however it did not reach statistical significance (Figure 4D). An analysis of gene markers in pMAT showed no change in the expression of *Fabp4* and *Adip*, as well as beige markers (Figure 4E). Similarly, no changes in the profile of dMAT gene expression were observed. In summary, testosterone deficiency had a wasting effect on peripheral but not on marrow fat which showed a tendency to increase in volume suggesting that androgens control MAT volume differently than the volume of peripheral fat depots. However, an absence of gonadal hormones in males did not alter the expression of metabolic gene markers in pMAT.

## Discussion

The presented studies are a continuation of characterization of MAT activities in physiological and pathological conditions. They have been designed based on previous observations that MAT is characterized by significant plasticity, and that its metabolic profile follows changes in systemic energy metabolism in conditions of aging and diabetes, and that MAT of beige characteristics secretes bone anabolic activities which may support bone remodeling in endocrine/paracrine manner (7, 11, 12, 14). Here, we extended our analysis by comparing MAT phenotype-specific gene markers profile in different locations within tibia bone and with respect to the presence of sex steroids in males and females. We used MAT of tibia bone as a model because of its unique distribution which correlates with the presence and the absence of bone remodeling process.

First, we have showed that pMAT, which is juxtaposed to the remodeling bone, consists of adipocytes dispersed between trabeculae. In contrast, MAT in distal tibia, where bone remodeling is practically absent, consists of densely packed osmium tetroxide-stained adipocytes occupying entire marrow cavity and being in physical contact with the endosteal bone surface. This distribution correlated with the profile of gene markers expression which appeared to be more beige-like in proximal when compared with the distal location. In this case, we have applied expression patterns of gene markers previously identified as specific for either white, or brown, or beige adipocytes in peripheral fat depots (15–17). Our studies represent first attempt to apply biomarkers identified in peripheral adipocytes for phenotyping bone marrow adipocytes. We have showed, that MAT does not express BAT-exclusive *Zic1* marker, and WAT-exclusive *Tcf21* marker, and beige-specific *Tmem26* marker, suggesting different phenotype of marrow adipocytes from peripheral adipocytes. Moreover and as we showed previously, the expression of *Ucp1*, although detectable, is relatively low when compared with other fat depot (11), as well as expression of *Hoxc9*, indicating that marrow adipocytes differ from classical brown and beige adipocytes (not showed). At the same time, the expression of other brown/beige markers, such as *Prdm16, Tbx1*, and *Dio2*, was at the relatively high levels and showed differential regulation in pMAT vs dMAT, and in males vs females.

Second, we showed that high BV/TV correlates inversely with MAT volume in the same locations in a sex-specific manner. In age-matched 4-month old C57BL/6 mice, pMAT volume was 10-fold lower while BV/TV was 50% higher in males vs females. Interestingly, dMAT volume, as well as CtTh measured in close proximity to dMAT, did not differ between sexes. However, pMAT volume correlated inversely not only with BV/TV, but also with relative increase in the expression of beige fat markers. This together with our previous observation that marrow adipocytes of beige phenotype secrete bone anabolic factors support attractive hypothesis that the metabolic profile of MAT contributes to the regulation of bone mass, and by proxy, that MAT can be a therapeutic target to increase bone mass.

Third, we have tested whether MAT volume and its phenotype are under gonadal control. To this end, we have showed that MAT volume is under a negative control of ovary hormones. In the absence of ovaries, MAT volume was significantly increased in both proximal and distal location. We attributed the expansion of pMAT rather to *de novo* differentiation of adipocyte progenitors, as indicated by increased expression of *Fabp4* and *Adip*, than increase in volume of already existing adipocytes which may constitute a characteristic response of dMAT and gWAT to estrogen deficiency. Indeed, this would be consistent with previous observation that peripheral fat depots respond to the absence of estrogen with increased lipogenesis but not adipocyte differentiation (20). However, it can be debated whether relative decrease in beige markers expression in pMAT suggests a direct effect of estrogen on beige adipocyte phenotype or a relative decrease of beige adipocyte fraction as a result of increased fraction of newly differentiated white-type adipocytes. Solving these possibilities requires further investigation. In contrast, a loss of testes led to a loss of peripheral WAT and BAT mass, but did not affect and even modestly increased MAT volume suggesting that MAT, unlike peripheral fat, is rather resistant to the effects of androgens deficiency.

Most importantly, we did not observe in both models that bone loss due to the absence of gonadal hormones is coupled to MAT expansion in the same location. Thus, it is possible that the targets and mechanisms by which sex steroids control bone mass are different from those which control MAT expansion leading to the conclusion that unlike in other models, e.g., aging, diabetes, or treatment with antidiabetic TZDs, MAT may not play a significant role in the bone loss and its expansion is merely a byproduct of sex steroids deficiency. However, MAT expansion and changes in the metabolic profile toward lipid accumulation and away from lipid utilization may contribute to bone loss via increased lipotoxicity and increased pro-inflammatory signaling in the marrow environment. Interestingly, it has been recently demonstrated that MAT contains a population of PTHr1-positive adipocytes which have a capacity to secrete RANKL cytokine (30). Perhaps, it is provocative at this point to hypothesize that sex steroids deficiency increases pool of RANKL producing adipocytes which may significantly contribute to the increased bone resorption and bone loss. Such possibility requires further testing.

The presented studies carry certain limitations and caution should be applied in the interpretation of the results. The analysis of gene markers was conducted using RNA isolated from either proximal or distal half of tibia. These isolates, especially from proximal tibia, were characterized with high cellular heterogeneity and our results required normalization to the expression of adipocyte-specific gene markers *Fabp4* and *Adip*, whose expression we assumed was relatively similar in pMAT and dMAT adipocytes. Such experimental design was applied previously (11) and also chosen for these studies because a very limited quantity of adipocytes in the murine proximal tibia prohibits isolation of adipocyte fraction suitable for analysis. Moreover, not all cells of adipocyte lineage with endocrine activity contain lipid droplets. We consider this as a limitation of our studies which results should be interpreted rather as a relative comparison. Recently, significant advances have been made in characterization of marrow adipocyte lineage, which appears to be closely related to the osteoblast lineage based on an expression of osteoblast gene markers such as *Osterix* and *Prx1* (30, 31). We believe, that these findings will result in a development of reliable method of marrow adipocyte identification and with a specific lineage tracing we will be able to follow changes in their phenotype in physiological and pathological conditions.

In conclusion, MAT is a heterogeneous tissue which may play different functions in bone depending on its location and metabolic phenotype. This heterogeneity suggest that MAT may play a role in regulation of bone remodeling and may contribute either beneficially to the maintenance of bone homeostasis, e.g., in “healthy” conditions it may support bone remodeling by providing energy and perhaps bone anabolic cytokines, or negatively in conditions of “unbalanced” bone remodeling, e.g., during aging which skews marrow mesenchymal cells toward adipocyte and at the expense of osteoblast differentiation. However, our studies argue against direct association between the mechanisms by which sex steroids control MAT expansion and bone mass, although indirect effect on modulation of marrow environment is not excluded. Moreover, these studies emphasize MAT uniqueness, when compared with peripheral fat depots, in respect to gene markers expression and response to hormonal control by sex steroids.

## Ethics Statement

This study was carried out in accordance with the recommendations of University of Toledo Health Sciences Campus Institutional Animal Care and Utilization Committee. The protocol was approved by the Institutional Animal Care and Utilization Committee.

## Author Contributions

BL-C developed hypothesis, designed experiments, analyzed and interpreted results, and wrote manuscript. LS performed experiments, analyzed data, and contributed to writing. PC mCT analysis of bone and fat volume, performed experiments, analyzed data, and contributed to writing. SS, SH, and AK performed experiments and analyzed data.

## Conflict of Interest Statement

The authors declare that the research was conducted in the absence of any commercial or financial relationships that could be construed as a potential conflict of interest.
